# The experiences of transgender and nonbinary adults in primary care: A systematic review

**DOI:** 10.1080/13814788.2023.2296571

**Published:** 2024-01-10

**Authors:** Daisy Holland, Luka C. J. White, Marija Pantelic, Carrie Llewellyn

**Affiliations:** a Brighton and Sussex Medical, University of Sussex, Brighton, UK; b Independent Transgender Community Researcher, Brighton, UK

**Keywords:** Systematic review, transgender, primary care, healthcare access

## Abstract

**Background:**

Transgender and nonbinary (TNB) people face barriers to primary care, which remains the main entry point for accessing gender-affirming healthcare in the UK.

**Objectives:**

This systematic review aims to summarise the evidence regarding TNB people’s experiences of primary care to inform improvements in service and patient outcomes.

**Methods:**

This review followed the Preferred Reporting Items for Systematic Reviews and Meta-Analyses (PRISMA) reporting guidelines. A systematic literature search was conducted across articles from 2005 to April 2023 across Ovid Medline, Ovid Embase and PsychInfo using established keywords relating to gender identity, primary care and experiences. Qualitative data were thematically analysed and quantitative data were compiled using a descriptive narrative.

**Results:**

Following eligibility criteria, 16 articles were included in this review. This review identified both facilitators and limitations and barriers experienced by TNB people related to primary care provider knowledge; the patient-provider relationship, and healthcare settings. Quantitative findings reported up to 54.4% of participants were uncomfortable discussing TNB issues with their physician. Overall findings suggest TNB people face discrimination on a systemic level utilising primary care services, though positive healthcare encounters at a local level were reported. Participants expressed a desire for primary care-led gender-affirming healthcare services, with involvement from local TNB communities.

**Conclusion:**

This review demonstrates TNB people’s mixed experiences of primary care alongside their recommendations for service improvement. This is the first systematically reviewed evidence on the topic, emphasising the need for clinicians and policymakers to centre the voices of the TNB community in service design and improvement.

## KEY MESSAGES

Many TNB participants had negative experiences in primary care, attributed to a lack of healthcare provider knowledge and medical gatekeeping.

Many TNB participants desired gender-affirming healthcare services to be more primary care-led.

Primary care services should consult local TNB communities in service design, evaluation and improvement.

## Introduction

Transgender (trans) is an umbrella term to describe those who go across or beyond culturally defined categories of gender [1]. In European culture, or cultures with a history of European colonisation, gender is culturally defined as binary, comprising two mutually exclusive categories (man or woman), which are assigned at birth based on the observable biological sex traits of the infant [2]. Individuals who have the binary gender they were assigned at birth are described as cisgender (cis man or cis woman). Trans individuals may have a binary gender different to their assigned gender at birth (trans man or trans woman), or they may not have a binary gender (nonbinary person). Trans and nonbinary (TNB) people make up a minority group and are marginalised in these cisnormative cultures [1,2]. Cisnormativity is the assumption that being cisgender is normal, and that individuals will generally conform to the binary gender norms of that culture in that time [2].

In the United Kingdom (UK), with increased sociocultural awareness of TNB identities, primary care services are more likely to care for TNB patients. However, across healthcare services broadly, TNB people experience poorer physical and mental health outcomes compared to the cisgender population, which in part may be due to the direct and indirect effects of stigma, transphobia, and the general cultural climate of hostility towards TNB people globally [3]. This is encompassed under the minority stress model, which describes increased stresses faced by marginalised people due to institutional stigma and discrimination and the impact of such on individuals’ health and well-being [4].

Within primary care, TNB people commonly have adverse experiences. Examples of this include general practitioners (GP) being unwilling to provide or support TNB people in accessing gender-affirming healthcare (GAHC), such as hormone replacement therapy (HRT) and/or gender-affirming surgery [5–7]. In the UK, TNB people who wish to medically transition often first try to access GAHC through their GP as part of the National Health Service (NHS) GAHC pathway [8]. The GP can make a referral to the Gender Identity Clinic (GIC), where individuals are assessed by a psychiatrist for a diagnosis of gender dysphoria, which is necessary for further state-funded interventions, such as HRT [8].

These pathways rely on healthcare professionals’ assessment of the patient’s gender identity. This often results in TNB individuals’ experiencing pressure to conform to cisnormative expectations of presentation and behaviour to access care [6,9,10]. There are added difficulties for nonbinary people, as healthcare services and providers may have a binary view of gender, which contributes to nonbinary individuals feeling ‘invisible’ in navigating both trans-specific and broader healthcare services [11]. These barriers impact primary care services, such as cancer screenings, where research suggests there is lower uptake by TNB people partially attributed to experiences of discrimination [12].

Moreover, there are significant waiting times to access a GIC, which has a significant impact on individuals’ mental health. The UK Trans Mental Health Study found that 63% of TNB participants reported increased suicidal ideation before transitioning, which decreased to 3% after accessing GAHC [10]. To avoid delays, many TNB people access GAHC through private providers instead. Associated financial stresses and the mental health impact of this may influence both TNB people’s broader health and further experiences within primary care.

To our knowledge, a systematic review of TNB people’s experiences within primary care is lacking. Existing studies have tended to examine aspects of TNB mental health [6,13,14] or general healthcare [15,16]. Primary care-focused studies largely centre on TNB youth [17–19]. There is a need to better understand TNB adults’ experiences of primary care, so as to inform improvement of primary care services, where a growing number of TNB people present to their GP. Within cisnormative cultures, GPs and primary care providers may experience challenges in providing or facilitating access to GAHC; however this is not the focus of this review. This review asks: What are TNB adults’ experiences of primary care globally?

## Methods

A study protocol was registered at the International Prospective Register of Systematic Reviews (PROSPERO) with a Registration number CRD42022330310 and is available from (https://www.crd.york.ac.uk/PROSPERO/?msclkid=8c7ec6a6d13c11eca8a7c1709e55f422#myprospero). The systematic review follows the Preferred Reporting Items for Systematic Reviews and Meta-analysis (PRISMA) guidelines (Supplementary material) [20].

### Search strategy and selection criteria

A systematic search was performed to identify quantitative and qualitative studies regarding the experiences of TNB people in primary care. Keywords and Medical Subject Heading (MeSH) terms were developed and combined using (AND) and (OR) Boolean operators. The search was limited to articles from 2005 to April 2023. This was chosen for two main reasons: Prior to 2005, the Gender Recognition Act (GRA) 2004 was not enforced, which in part shaped the way primary care acted as the entry point to specialist gender identity services in the UK [21]. Additionally, the development of GAHC pathways and services were generally limited globally. No limits were set on study location as this field has limited literature. Only studies written in English were screened. Data collection and screening were conducted from 30th June to 18th August 2022 by one independent reviewer and again in full between 11th and 17th April 2023 by two independent reviewers. A third reviewer resolved any disputes concerning which studies were eligible for inclusion. Three databases were used: Ovid Medline, Ovid Embase, and PsychInfo. This search was further supplemented by searching the reference lists of relevant studies.

Population search terms were: transgender OR trans OR gender non-conforming OR nonbinary OR gender diverse OR gender identity OR transsexual. Exposure-related search terms were: primary healthcare OR primary care OR general pract* OR GP OR healthcare service* OR healthcare access OR trans* inclusive. Outcome-related search term included: Experience* OR perception* OR expectation* OR marginalisation OR transphobia OR accessibility OR barrier* OR facilitator* OR attitude* OR stigma. Outcome specific search terms were kept broad to ensure the relevant literature could be screened whilst maintaining focus on capturing TNB people’s voices. The titles and abstracts were screened, removing any irrelevant and/or duplicate studies. The remaining studies were assessed for eligibility per the inclusion and exclusion criteria (Table 1). A full-text review of eligible studies was then performed.

**Table 1.** Inclusion and exclusion criteria.

Inclusion Criteria	Studies focusing on TNB^a^ people
	Studies focusing on experiences or interactions with general practice, primary care, family physicians, general practitioners or family medicine
	Primary research studies including quantitative, qualitative and mixed methods methodologies
	Any country for study location
Exclusion Criteria	Editorials, opinion pieces and non-primary data
	Studies published not in English
	Publication date prior to 2005
	Duplicate study
	No mention of TNB people as a population or participant focus
	Studies with a specific healthcare focus other than primary care (e.g. secondary or tertiary care services, social services)
	Studies focusing on TNB youth below age 18 years

^a^ TNB: transgender and nonbinary.

### Data extraction and synthesis

Key information was extracted and summarised to compare different study characteristics and outcomes. Thematic analysis was used to synthesise themes from the qualitative studies included in this review, following Braun and Clarke’s six-step process [22]. This involves familiarising data, generating codes and searching, analysing and defining recurrent themes and patterns. Each study was read multiple times, with key themes and concepts recorded to formulate patterns between studies to group overall themes. Regarding quantitative studies, a narrative synthesis was completed whereby relevant data was compiled in a table with an accompanying narrative summary presented.

### Quality assessment

The Mixed Methods Appraisal Tool (MMAT) was used to appraise the quality of the study reporting [23] (Table 2). Quality appraisal was performed by two independent reviewers, both of whom had been trained in using the quality appraisal tool.

**Table 2.** Quality assessment of sixteen included studies using MMAT [23].

Study Design	Author, year of study	Methodological study criteria				
		Is the qualitative approach appropriate to answer the research question?	Are the qualitative data collection methods adequate to address the research question?	Are the findings adequately derived from the data?	Is the Interpretation of results sufficiently substantiated by data?	Is there coherence between qualitative data sources, collection, analysis and interpretation?
Qualitative	Wright et al. 2021 [24]	Yes	Yes	Yes	Yes	Yes
	Haire et al. 2021 [25]	Yes	Yes	Yes	Yes	Yes
	Ker et al. 2020 [26]	Yes	Yes	Can’t tell	Yes	Can’t tell
	Willis et al. 2020 [27]	Yes	Yes	Yes	Yes	Yes
	Allory et al. 2020 [28]	Yes	Yes	Yes	Yes	Yes
	Bell and Purkey 2019 [29]	Yes	Yes	Yes	Yes	Yes
	Zwickl et al. 2019 [30]	Yes	Can’t tell	Yes	Can’t tell	Yes
	Vermeir et al. 2017 [31]	Yes	Can’t tell	Yes	Yes	Yes
	Westerbotn et al. 2017 [32]	Yes	Yes	Yes	Yes	Yes
	Melendez et al. 2009 [33]	Yes	Yes	Yes	Yes	Yes
Quantitative descriptive		Is the sampling strategy relevant to address the research question?	Is the sample representative of the target population?	Are the measurements appropriate?	Is the risk of nonresponses bias low?	Is the statistical analysis appropriate to answer the research questions?
	Treharne et al. 2022 [34]	Yes	Can’t tell	Yes	Yes	Yes
	Kattari et al. 2021 [35]	Yes	Can’t tell	Can’t tell	Yes	Yes
	Bauer et al. 2015 [7]	Yes	Can’t tell	Yes	Can’t tell	Yes
Mixed methods		Is there an adequate rationale for using a mixed methods design to address the research question?	Are the different components of the study effectively integrated to answer the research question?	Are the outputs of the integration of qualitative and quantitative components adequately addressed?	Are divergences and inconsistencies between quantitative and qualitative results adequately addressed?	Do the different components of the study adhere to the quality criteria of each tradition of the methods involved?
	Tan et al. 2022 [36]	Yes	Yes	Yes	Yes	Yes
	Goldenberg et al. 2021 [37]	Yes	Yes	Yes	Can’t tell	Yes
	Hinrichs et al. 2018 [38]	Yes	Can’t tell	Yes	Can’t tell	Yes

Both screening questions were positive for all included studies: 1) Are there clear research questions? (2) Do the collected data allow to address the research questions?

## Results

**Figure 1.**
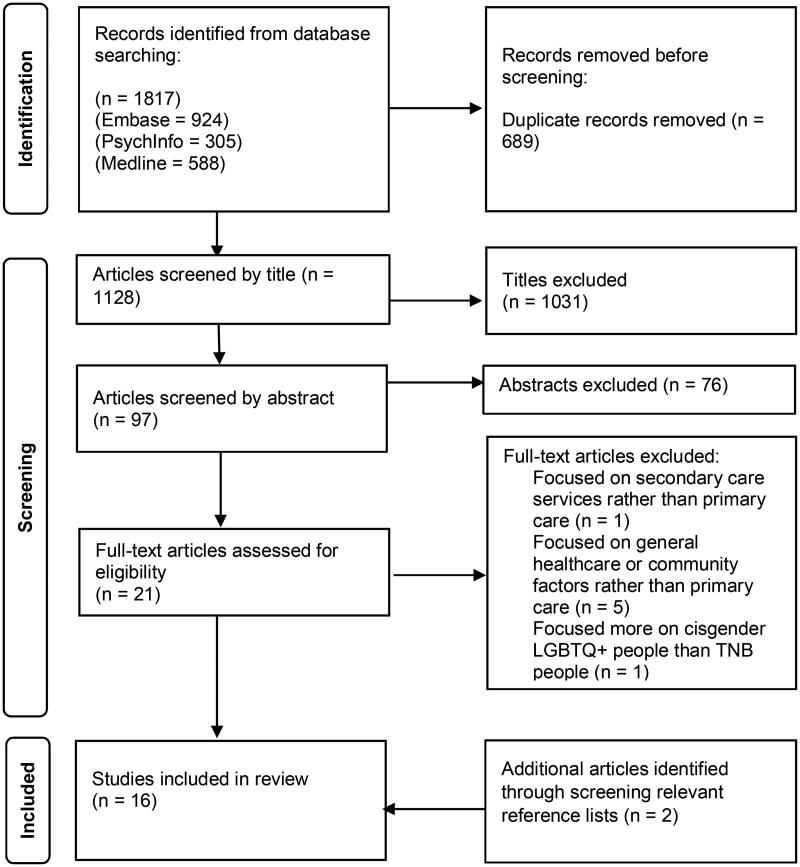
PRISMA diagram showing study selection process.

The search strategy yielded 1,817 identified titles, with 1,128 titles screened for eligibility after removing duplicate studies. From these titles, 97 abstracts were screened with a full-text screening of 21 articles (Figure 1). The 16 studies included are summarised in Table 3.

**Table 3.** Summary of the 16 included studies.

Author, year, country	Study type	Participant characteristics	Experiences and perceptions of primary care	Expectations from a primary care service/healthcare providers	Key findings
Wright et al. 2021, UK, [24]	Qualitative study, interview	Transgender people who had also taken part in the HIV Self-Testing Public Health Intervention (SELPHI) randomised control trial Total participants − 20 Gender − 7 trans women, 12 trans men, 1 nonbinary trans masculine person Ages − 21–57	Examination of the pathway from primary care to specialist gender identity services in the UK	NR	GPs are poorly informed about TNB identities and care pathways Cisnormative views enforcing the gender binary may alter how patients present to primary care in order to access care Positive interactions with GPs are when they prioritise treating the patient holistically as a whole person and have a willingness to learn and take responsibility
Haire BG et al. 2021, Australia, [25]	Qualitative study, interview	TNB people living in Australia, recruitment facilitated by the Gender Centre Total participants − 12 Gender − 9 trans women, 1 trans man, 2 nonbinary people Ages − 19 to 59 Race/Ethnicity − 5 participants were Indigenous	Experiences of healthcare access in trans and gender diverse people with complex health needs	NR	TNB people have to navigate multiple HCPs and HCS and HCPs act as gatekeepers Perceived erasure of nonbinary identities and a need to conform to gender stereotypes to access care Perceived ignorance and stigma from HCPs common A welcome physical environment and supportive peer community seen as vital
Ker et al. 2020, New Zealand, [26]	Qualitative, interview	TNB people accessing a primary care pilot clinic providing HRT Total participants − 4 TNB people and 4 HCPs Gender – of the service users: 3 female, 1 nonbinary and neutrois Ages − 18-26	Exploring the experiences of a primary care-based pilot clinic providing gender-affirming hormone therapy in New Zealand	NR	Primary care led clinics were perceived as more accessible, with less waiting times and less gatekeeping HCPs emphasised partnership with service users and that the service provided was adaptable Perception it depathologised gender diversity Efforts to centralise care seen as minimising the power dynamic
Willis et al. 2020, Wales, [27]	Qualitative, interview	Older TNB people who were trying to medically transition later in life Total participants − 19 Gender − 15 trans women, 4 trans men Ages − 50 to 74	Examining supportive and obstructive points of interaction with health-care professionals	NR	GPs were inconsistent allies Participants often forced into the role of patient educators as GPs lacked knowledge of trans needs, available treatments and care pathways Perception HRT could be GP managed but seen as too specialist Some GPs would act as individual gatekeepers
Allory et al. 2020, France, [28]	Qualitative interview	TNB adults in France recruited through local trans or LGBTI (lesbian, gay, bisexual, trans, and/or intersex) associations, primary care providers, and social networks Total participants − 27 Gender − 14 trans men, 12 trans women, 1 nonbinary person Ages − 18 to 60	Exploring the difficulties experienced by transgender people in accessing primary health-care services	Exploring expectations towards primary care providers to improve their health-care access.	Nano, micro, meso and macro levels of difficult accessing primary care services Anxiety in anticipating GP responses and being “outed” in waiting rooms TNB people form peer community networks and refer on trusted HCPs Healthcare systems not adapted to TNB people Expectations of gender normativity common and pressure to conform to gender stereotypes to access care Expectations included self-determination and reducing the need for psychiatric assessment TNB people want primary care led services for initiating and following up treatments, perceived to be more holistic and accessible
Bell et al. 2019, Canada, (29)	Qualitative, interview	TNB people in Ontario recruited through family medicine clinics and community agencies Total participants − 11 Gender − 4 trans men, 6 trans women, 1 GNC	Exploring past experiences of TNB people in primary care	Describing expectations of members of the trans community regarding the delivery of primary care by their family physicians	Perceived HCP knowledge of TNB identities found lacking overall and limited knowledge about hormones Patient self-advocacy beyond what would be expected, e.g. bringing in guidelines or referring GPs to local events Discrimination by HCPs common Primary care led treatment preferred as perceived to be more holistic though more education is needed More research into the systemic oppression of trans people which has led to an intersectionality with poverty, mental illness and substance abuse
Zwickl et al. 2019, Australia, [30]	Qualitative, survey	TNB adults in Australia recruited through the trans medical research Facebook group Total participants − 928 Gender − 37% trans women or trans femme, 36% trans men or trans masc, 27% nonbinary identities	Exploring health needs of trans and gender diverse people in Australia	NR	80% have a regular HCP Mainstreaming services to primary care will likely improve accessibility TNB people should guide research priorities and health service delivery 44.8% said HCP knowledge lacking Positive interactions including respecting names, pronouns, not asking invasive questions and knowing about care pathways Need for more research about HRT risks long term and about improving care
Vermeir et al. 2017, Canada, [31]	Qualitative, interview	TNB adults living in Nova Scotia who wanted to experience, or had tried to experience, primary and/or emergency care within the last two years Total participants − 8 people Gender − 3 trans women, 1 trans man, 1 trans masc guy, 1 transsexual man, 1 genderqueer and nonbinary person, 1 dude/guy/demi-guy/person Ages − 18 to 44	Exploring the barriers trans adults encounter when pursuing primary and emergency care in Nova Scotia, Canada		Interpersonal barriers to care included HCP knowledge and patients having to educate HCPs Reasons as to why patients seeking care taking a backseat Perceived uncomfortable attitudes by HCPs, including deadnaming, being “outed”, “gendering” care, invasive questioning Lack of welcome physical spaces e.g. gender neutral bathrooms, exclusion of TNB inclusive information on posters and forms Extensive wait times for specialist care
Westerbotn et al. 2017, Sweden, [32]	Qualitative interview	TNB people living in Stockholm with experience of the Swedish healthcare system Total participants − 14 Ages − 20 to 50	Experiences of TNB people meeting with healthcare professionals	Exploring expectations TNB have before and upon meeting HCPs	Clear knowledge gap among HCPs TNB people wanted to be treated like anyone else TNB people often expected ignorance or discrimination Many participants felt anxious before meeting with HCPs which led to avoidance of services of fear of being exposed / questioned by HCP and reception staff
Melendez et al. 2009, USA, [33]	Qualitative, interview	Trans women who took part in a study around their ideas of best practice for HIV prevention and primary care in a specific community-based clinic in New York City. Total participants − 20 Gender - all trans women Ages − 18 to 53 Race/ethnicity − 16 were Latina (14 Puerto Ricans, 1 Central American, one South American), 4 were African American	Exploring the experience of trans women at this particular primary care and HIV prevention clinic, which was not an LGBT-specific clinic.	Exploring what trans women think best practice looks like for providing primary care and HIV prevention support to trans women in a community clinic.	Some participants preferred using a general health clinic to an LGBT specific clinic because they saw themselves as women, rather than transgender women, and because of tensions within the LGBT community. The participants reported feeling safe at the clinic and many had been recommended the clinic by friends as it had been reported to be safe. Factors influencing their feelings of safety included being called by their preferred names, feeling cared for, and understood their community, including the intersection of their (primarily Latinx) racial/ethnic identity and their identity as trans women. They also reported feeling like the clinic could provide a ‘one stop shop’ for medical care, sexual healthcare and gender affirming care.
Treharne et al. 2022, New Zealand, [34]	Quantitative, survey	TNB people living in New Zealand with a primary care doctor or GP Recruited through social networks and within local trans and queer communities Total participants − 948 Gender − 29% trans women, 29% trans men, 42% nonbinary identities Ages − 14 to 83	Experiences of the most common positive and negative experiences with primary care doctors for TNB people	NR	Supportive experiences include equitable treatment from doctors and cultural competence Negative healthcare experiences associated with distress and non-suicidal self-injury and suicidality 47% had to educate healthcare providers, 28% were told gender-affirming care could not be provided on account of lack of knowledge 48% had primary care doctors supportive of their healthcare needs Nonbinary people more likely to have negative healthcare experiences in accessing gender affirming care
Kattari et al. 2021, USA, [35]	Quantitative, cross-sectional survey	Transgender Adults (18+) in the US, recruited online. Total participants − 27,715 Gender − 28.6% man/ trans man, 33.85% woman / trans woman, 34.15% nonbinary/genderqueer, 2.62% cross-dresser = 2.62% Age: *M* = 31.23 Ethnicity − 80.76% white/European American, 5.24% Latinx/Hispanic, 4.65% Biracial/Multiracial, 2.79% Black/African American, 2.52% Asian/Asian American, 1.07% American Indian, 2.96% Other	To assess how many TNB individuals see a physician on a yearly basis; how many TNB individuals have a regular primary care provider (PCP); and how many of these individuals have a PCP who they would consider to be knowledgeable about transgender health	NR	17.8% reported that their PCP knew almost nothing about TNB health, 17.7% that their PCP knew some things, 12.6% that their PCP knew most things, and 15.2% that their PCP knew almost everything. The remaining 36.7% did not know how much their PCP knew about TNB health. Being nonbinary/genderqueer or a crossdresser was associated with lower levels of PCP knowledge, being a trans woman was associated with higher levels of PCP knowledge.
Bauer et al. 2015, Canada, (7)	Quantitative, cross-sectional survey	Transgender people in Canada, recruited online using respondant-driven sampling. Total participants − 433 Gender − 184 (52%) were assigned female at birth (AFAB), 172 (48%) were assigned male at birth (AMAB) Ages − 16 to 25 = 41% TM 20.9% TF; 25-45 = 48.8% TM, 47.6% TF; 45 + 10.2% TM, 31.5% TF. Race/ethnicity − 68.1% were white, 31.9% were racialised or Aboriginal.	To assess how comfortable participants are to discuss trans healthcare with their PCP (Discomfort/Comfort). Checklist of 9 trans-specific negative experiences with GPs. Provider knowledge of trans issues, 4-point Likert scale.	NR	47.7% AFAB trans people and 54.5% AMAB trans people were not comfortable discussing trans issues with their doctor. This discomfort was present despite accounting for factors such as access to a regular family physician and having universal healthcare insurance. Having a provider perceived as knowledgeable was associated with more comfort, and previous negative experiences were associated with more discomfort. Higher levels of transphobia were independently and negatively associated with discomfort among transfeminine participants.
Tan et al. 2022, New Zealand, [36]	Mixed methods, cross-sectional survey with quantitative and qualitative outcome variables	Trans people (aged 14+) living in New Zealand who are accessing primary care. Total participants – Quantitative *n* = 871, qualitative *n* = 153 Gender – Quantitative: 29.1% trans women, 29% trans men, 41.9% nonbinary Qualitative: 12.3% trans women, 20.1% trans men, 33.3% nonbinary Ages – Quantitative: 15.3% aged 14–18, 28.1% aged 19–24, 34.6% aged 25–39, 13.4% aged 40–54, 8.3% aged 55+. Qualitative: 7.5% aged 14–18, 10.1% aged 19–24, 21.3% aged 25–39, 19.8% aged 40–54, 27.3% aged 55+. Race/ethnicity – Quantitative: 84.3% New Zealand/European, 12.9% Maori, 1.5% Samoan, 1.4% Chinese. Qualitative: 18.3% Maori, 16.6% NZ European, 10.3% other.	To assess participants confidence in their GP in explaining information, involving them in decision-making and consideration of financial factors To explore broader experiences in primary care	NR	Transgender participants had greater risk of feeling no confidence in their GPs (Cohen’s *d* = 0.39), reporting barriers accessing primary care due to cost (38.4% vs 17.4%; RR = 2.21), and transport issues (13.5% vs 3.0%; RR = 4.58) compared to the general population Enabling resources relating to affordability of care and transport were key factors in accessing primary care, with regional variability comparing suburban to rural areas.
Goldenberg et al. 2021, USA, [37]	Mixed methods, cross-sectional survey and individual in-depth interviews	Trans youth of colour living in 14 US cities who had experienced using healthcare. Survey, total *N* = 79 Interviews, total *N* = 33 Gender – Trans femme = 20, Trans masc = 13. Ages − 16 to 24 Race – Non-Hispanic Black = 14, Asian/Pacific Islander = 4, Latinx/Hispanic = 11, Multiracial = 4.	To explore quantitatively the need for, and access to, gender affirmation within healthcare To qualitatively explore healthcare experiences, and barriers and facilitators to accessing care.	NR	Finding a provider was described as challenging, although some had positive experiences due to being referred to specific affirmative providers *via* other services or through recommendations from within the trans community. They described barriers in making an appointment and being in the waiting room. Participants reported positive and negative healthcare experiences, according to provider knowledge, whether they were treated with respect, and how much support they were given to navigate the healthcare system. They reported more negative experiences as being linked to healthcare avoidance Healthcare avoidance was associated with providers using inappropriate words for body parts, not having gender neutral bathrooms, and not being able to access referrals
Hinrichs, 2018, USA, [38]	Mixed methods	TNB participants who have accessed TNB-related and/or primary care at Smiley’s family medicine clinic in Minnesota Total participants − 22 Gender − 4 transgender man, 4 transgender man/male, 3 transgender women/female, 3 female, 1 transgender woman, 1 transgender male, 1 transgender man/other, 1 trans man/genderqueer/GNC, 1 trans woman/other, 1 GNC transmasc bigender, 1 GNC/other Ages − 18 to 65	To explore how primary care clinics can improve care for TNB patients	NR	Negative experiences of misgendering, stereotypes, stigma and/or rejection of services from HCPs TNB people need time to build trust and HCPs need to be willing to learn Need for sensitive and inclusive HCPs where patients can seek care for non-transition related care as well where their identity isn’t the focus Patients would prefer HRT to be primary care led and wanted more research into the long-term impact of HRT Challenges of mainstreaming TNB competent primary care services included inconsistency of HCP education, reluctance to adapt and struggles to ensure clinic wide consistency

NR: not relevant; TNB: transgender and nonbinary; GP: General practitioner; HCP: healthcare provider; HCS: healthcare system(s); GNC: gender non-conforming; LGBTQ+: lesbian, gay, bisexual, transgender, queer and plus; HRT: hormone replacement therapy; PCP: primary care provider; AFAB: assigned female at birth; AMAB: assigned male at birth.

Ten of the studies were qualitative [24–33], three were quantitative [7,34,35], three were mixed methods designs [36–38]. All studies were conducted in high-income countries and published between 2009 and 2022: the USA [33,35,37,38], the UK [24,27], Australia [25,30], Canada [7,29,31], New Zealand [26,34,36], France [28] and Sweden [32].

One study included data solely from transgender women [33]. Nonbinary identities were under-represented across the studies; three studies had a majority of nonbinary participants [34–36]. Participant numbers ranged from 8 to 27,715. Participant ages ranged from 14 to 83, with four studies including data from participants under the age of 18 [7,34,36,37]. Only data pertaining to TNB adults above 18 years of age were included in the analysis.

Most studies were considered to have a good or satisfactory quality appraisal level. Table 2 shows the quality appraisal on included studies using the MMAT. Table 4 provides a descriptive assessment as to the quality of individual studies. Cohen’s kappa coefficient was calculated based on MMAT scoring to assess the level of agreement and interrater reliability between reviewers. This was calculated as 0.61, demonstrating substantial agreement between the reviewers as to the quality of the studies.

**Table 4.** Descriptive quality appraisal of included studies.

Study design	Author	Description
Qualitative	Wright et al. 2021 [24]	Clear qualitative aims and the qualitative approach and method were appropriate (semi-structured interviews). Data collection was adequate to address the research question. Qualitative data collection and analysis were described (thematic analysis). Quotes used to justify themes were adequate.
	Haire et al. 2021 [25]	Clear qualitative aims and the qualitative approach and method were appropriate (semi-structured interviews). The recruitment strategy could have been expanded to include more networks. There was limited discussion of the potential bias of recruiting participants who may have had a prior relationship with an interviewer. Qualitative analysis described (reflexive thematic analysis). Quotes used to justify themes were adequate. Interpretation is substantiated by the data.
	Ker et al. 2020 [26]	Clear qualitative aims and the qualitative approach and method were appropriate (in-depth interviews). Participant recruitment was poorly described. Qualitative data collection and analysis were described in-depth (thematic analysis). Interpretation is substantiated by the data. There is coherency between data sources, collection, analysis and interpretation.
	Willis et al. 2020 [27]	Clear qualitative aims and the qualitative approach and method were appropriate (life-history and semi-structured interviews). Participant recruitment was well-described. Data collection and analysis were described in-depth (thematic analysis using the framework method). There is coherency between data sources, collection, analysis and interpretation.
	Allory et al. 2020 [28]	Clear qualitative aims and the qualitative approach and method were appropriate (semi-structured interviews). Participant recruitment, data collection and analysis were thoroughly described (reflexive thematic analysis in an inductive approach).
	Bell et al. 2019 [29]	Clear qualitative aims and appropriate qualitative design and methodology (qualitative phenomenological approach using semi-structured interviews). Data collection and analysis were clearly outlined (thematic analysis). The quotes used to justify the themes were adequate. However, the researchers discarded phrases interpreted as ‘important’ yet not representing a common experience. This could have been discussed further.
	Zwickl et al. 2019 [30]	Research aims are clear with an appropriate methodology and design. Participants’ demographics are described clearly by gender. There is no data on demographics such as age, ethnicity or socio-economic status. It is difficult to ascertain as to whether this sample is representative of the target population. Not stated as to whether the survey was pre-tested prior to data collection. Qualitative interviews may have been a more appropriate method of data collection. There are variations in the number of answers to the questions of focus for analysis. Reasons behind the non-response rate to specific questions were not given. Statistical analysis well-described.
	Vermeir et al. 2017 [31]	Clear qualitative aims and the qualitative design was appropriate (methods influenced by social constructivism, queer theory and Rhodes risk environment framework). Data collection and analysis thoroughly described (one-on-one interviews; analysis through the constant comparative method and framework analysis). There were clear links between data sources, collection, analysis and interpretation. However, the sample size was small so the research question may not have been best addressed.
	Westerbotn et al. 2017 [32]	Clear qualitative aims and a descriptive qualitative design was used. Data collection and analysis were detailed (qualitative inductive content analysis following in-depth interviews). Interpretation of results were supported by the data collected.
	Melendez et al. 2009 [33]	Clear qualitative aims and methodology (semi-structured interviews). Participant recruitment and data collection were well-described. There was a small sample size and potential selection bias though interpretation of results were supported by the data collected.
Quantitative	Treharne et al. 2022 [34]	Research aims are clearly established with an appropriate methodology and design. Context is clearly described. However it is unclear how representative the sample population is of the target population. The measurements are appropriate for answering the research questions. Reasons for non-response and exclusion of participants from the study were not made fully clear. It was difficult to ascertain risk of non-response bias.
	Kattari et al. 2021 [35]	The research aims are clear and associated variables are clearly described. Participant demographics are described clearly by gender, sexuality, race and socioeconomic status. Though the sample was large it was unclear if it was representative of the population. The measurements are appropriate for answering the research question.
	Bauer et al. 2015 [7]	The research aims are clear with an appropriate methodology and design. However, it is unclear how representative the sample population is of the target population and there is mixed data including results from under-18s within the cohort. There was limited discussion of bias and there was no information on the number of exclusions or non-response from participants.
Mixed methods	Tan et al. 2022 [36]	The rationale for a mixed-methods approach was clear with appropriate methodology. There were in-depth discussion of both quantitative and qualitative results and how they interlinked. The data was integrated and cohesive.
	Goldenberg et al. 2021 [37]	There were clear and appropriate mixed-methods design and methodology, integrating multiple frameworks. Both quantitative and qualitative results were discussed and integrated with each study component adhering to their respective quality criteria.
	Hinrichs et al. 2018 [38]	The rationale for a mixed-methods appraoch was somewhat unclear but the methodology seems appropriate. There was limited discussion around the quantitative results and how this may have impacted participants overall healthcare experiences and their answers in the qualitative section. More could have been done to integrate findings. The different study components adhere to the respective quality criteria.

The research aims and analysis of both qualitative and quantitative studies were clearly discussed. However, the quantitative studies were limited in discussing the quality of their data collection. The mixed methods studies were of varying quality. Of the three included studies, one failed to justify a mixed methods approach [38] and two could have better integrated their quantitative and qualitative findings [36,37].

### Study findings

Three key themes were identified; 1. Primary Care Provider Attitudes and Knowledge, 2. The Patient-Provider Relationship, and 3. Healthcare Settings. Themes 1 and 2 were discussed in thirteen studies each and theme 3 comprised of eight studies.

#### Primary Care Provider (PCP) attitudes and knowledge

PCP attitudes were a recurring theme across thirteen studies [7,24,25,27,29–32,34–38].

#### Upholding cisnormative views and stereotypes

Experiences of discrimination created anxiety among participants over not knowing how PCPs would respond to their gender identity (Table 5, Quotation 1). Across all study methodologies, participants (8–948) would avoid primary care services altogether, irrespective as to why they may need to be seeking healthcare (Table 5, Quotation 2) [29,31,32,34,37].

**Table 5.** Quotations from included studies.

Theme	Sub-theme	Quotation number	Quotation
1.0 PCP attitudes	Upholding cisnormative views and stereotypes	1 2 3	“[His PCP said they] ‘saw a trans man, he was really a man, he wasn’t like you, he really looked like a man (laughs) he had a beard and everything’ during an appointment.” (27) “There is a widespread perception that primary care does not have good skills for dealing with transgender people. I would rather wait a while and see if my health issues subside by themselves before I seek healthcare.” (28) “[I] feel very, like, trapped by my doctor into presenting a certain way and if I even deviate from that a little bit then I won’t have my options.” (24)
	1.2 PCP knowledge	4	‘She [GP] said to me, “Well, I don’t agree with the NHS paying for medication of this sort for people like you”. I was really taken aback by that…. With a very meek voice, I said, “I’m willing to pay for them”. “Right,” she says, “If you’re willing to pay for them.”’ (29)
2.0 The patient-provider relationship	2.1 Perceived Affirmation or Rejection of Participants’ Gender Identity	5	“It is gold that when I come in, we’re having a conversation as two people who have information about the issue in question. One from a medical perspective and one from actually living it, and they are equally valid. You have resources I don’t, I have resources you don’t, and we’re a team. I really value that we are totally equal partners in what we’re doing.” (37)
	2.2 Ongoing support and gaps in medical research	6	“Research is totally lacking. I think we’re ignored as a population. The healthcare offered doesn’t seem to be based on good evidence and it’s like we’re medical research subjects undergoing experimental treatment. Nobody knows what they’re doing.” (30)
3.0 Healthcare settings	3.1 The physical environment	7 8	Like some sort of sign that has a statement that wouldn’t mean anything to anyone except transgender people. So, like ‘people of all genders.’ … A trans person sees that and is like … ‘all genders? … are they on board?’ It’s nice and stealthy and it opens the door. … It’s just enough to start putting the barriers down, breaking down the walls, and be like ‘I feel safer already.’ (31) “If I don’t see like a sign or an Aboriginal flag, or something referring to Aboriginal health services, I kind of get worried ‘cause I just think about the demographic of the area.” (25)
	3.2 The desire for more primary care-led services	9 10	“That was useful, you know, having a place I was familiar with and someone I was familiar with there, and also just being able to come up to campus and do it. It was much easier I think than - hospitals are scary.” (26) “In an ideal world it would be really nice if your family doctor could do it directly because you know, like, it’s really hard getting [hormones]. So it’s nice to have it with somebody you have an actual relationship with all the time.” (29)
	3.3 Expectations of primary care services	11 12	“So finally, between a GP that I can see quite easily and an endocrinologist who will potentially be more expensive and less accessible.” (28) “I would love it if everyone would have the expertise to deal with it. I would love it. The realist in me says ‘Let’s start small.’ Starting with just the desire and the understanding to want to learn and to know where to go to get the answers. And be willing to take that journey. That would be the willingness. Let’s start with the willingness to not be afraid of taking a new patient when they say ‘Oh, by the way, I’m transgendered.’ You don’t want to hear click, zzzzzz. It’s not a big deal—say ‘Yeah, oh, sure’—whatever. Right. That’s—we need health care. We need basic-human-right health care. That’s all we’re asking. That’s it.” (29)

Qualitative studies with 33 participants reported common experiences of participants having to alter their gender presentation to access care without being challenged (Table 5, Quotation 3) [29,31,32].

Positive healthcare interactions were associated with increased engagement with services, better confidence in navigating other healthcare systems and significantly decreased levels of psychological distress [34,37].

#### PCP knowledge and attitude

Lack of PCP knowledge was a major theme [7,24,25,27,29–32,34–38], reported between 28 and 54.4% of participants across quantitative studies, which ranged from 433 to 27,715 participants [7,34,35]. One quantitative study (*n* = 27,715) found that nonbinary people generally perceived their PCP as being ‘less knowledgeable’ not just about TNB identities and trans-specific healthcare needs but also how changing social and political climates negatively impact on TNB people’s health [35].

Across multiple studies, with 28,851 participants, TNB people had low confidence in PCPs and perceived themselves as not being involved in healthcare discussions and shared decision-making [35–37]. Due to a lack of general PCP knowledge regarding trans-specific healthcare, many TNB participants were often forced into the role of patient educator. Participants ranged from 11 to 1024 across multiple studies reported being the primary source of information for their PCP regarding GAHC [24,25,27,29–31,36], often compiling information through peer networks and online sources in a process described as *‘exhausting emotionally’* [29]. Some perceived the lack of proactive learning as unprofessional and as an unwillingness to learn. Studies with a total of 987 participants recalled being asked invasive questions irrelevant to their healthcare, perceived as being largely due to the PCP’s curiosity, to the detriment of the participants’ health and personal boundaries [25,27,31,34]. The incidences of participants being asked inappropriate questions were also seen as a way of PCPs exerting power as participants often felt forced to answer (Table 5, Quotation 4) [25,27]. Limited knowledge regarding GAHC pathways leading to referral delays was frustrating, especially to older TNB participants who expressed anxiety about ‘losing time’ [27].

Positive experiences within primary care centred around having a proactive GP with an awareness of TNB specific and inclusive healthcare needs [24,25,27,30]. One study of 433 participants found having a knowledgeable PCP was associated with up to 63% decrease in levels of discomfort [7]. Another quantitative study showed participants (*n* = 948) who had a supportive, knowledgeable PCP were less likely to have attempted suicide over 12 months [34].

#### The patient – provider relationship

Topics relating to the patient-provider relationship came up across thirteen studies [7,24–31,33,36–38].

#### Perceived affirmation or rejection of participants’ gender identity

How well participants viewed their care was highly dependent on the extent to which their gender identity was affirmed or rejected. The most common rejection perceived by participants was through misgendering or deadnaming (referring to a TNB person by a name they used prior to transitioning) [27,29,33,37]. This includes a failure to update medical documentation, which further contributed to the issue of healthcare avoidance [25,27,31,32].

Positive examples of gender affirmation included PCPs being non-judgmental, using the correct name and pronouns and adapting the language they used for each patient, acknowledging them as an expert in their own self (Table 5, Quotation 5) [24,29,30,33,37,38].

#### Ongoing support and gaps in medical research

Largely qualitative studies, with a total of 1,090 participants, reported a lack of ongoing support from their GP at various points: when on waiting lists for GAHC; following gender-affirming treatment; and when accessing specific healthcare services associated with gendered anatomy, for example, reproductive cancer screenings [25–28,36]. This extended to a general perception within one qualitative study (*n* = 928) that medical research was neglectful of TNB populations (Table 5, Quotation 6) [30]. Specifically, 27% of participants expressed there was not enough research on the long-term effects of HRT and almost 19% of participants wanted further research into improving gender-affirming surgical techniques [30].

1. *Healthcare Settings*
  Healthcare settings were a theme across eight studies [25,28,29,31–33,37,38].

#### The physical environment

Participants across qualitative studies, ranging from 8 to 27, said that a welcoming physical environment was an important facilitator to seeking out primary care services. This included having visible posters or signs that could indicate safe spaces to TNB people (Table 5, Quotations 7–8) [25,28,29,31].

The waiting area was a key source of anxiety, primarily due to the perceived lack of privacy and fear of uncertainty as to whether participants would be misgendered by reception staff in front of others [28,29,31,32,37]. This was exacerbated for participants in rural areas or who were more socioeconomically disadvantaged; participants would travel significant distances to attend a specific primary care centre to avoid these stresses [28,36].

#### Desire for more primary care led services

Multiple studies, spanning five countries, with 87 participants, expressed a desire from TNB people for more primary care-led services providing GAHC, particularly HRT [26–29,38]. Primary care was viewed as more holistic and accessible, with less waiting time and reduced financial and travel costs. Participants from qualitative studies, ranging from 8 to 22, felt better able to make informed health choices due to alleviating additional logistical stresses [26,38]. Integrating services within primary care was suggested as a method of increasing patient autonomy, service accessibility and reducing medical gatekeeping (Table 5, Quotations 9–10) [26–29,38].

#### Expectations of primary care services

Three studies explored expectations of ideal care. Participants ranged from 27 to 928, and most participants wanted, at minimum, their PCP to have basic knowledge around different TNB identities, how to initiate HRT and how to access further resources [28–30]. One study detailed a desire by participants for practices to employ TNB people as advisors or educators to help inform practice policy and care delivery [30]. Ideal care centred around principles of self-determination, where TNB people could access GAHC and legal documentation without the need for psychiatric assessment (Table 5, Quotations 11–12) [28].

## Discussion

### Main findings

Findings from this review suggest that TNB people in high-income countries face insufficiently trained providers and discrimination when utilising primary care services. Primary care was perceived negatively on a systemic level, with some participants having more positive experiences on account of individual PCPs or clinics. Positive healthcare interactions were associated with increased engagement with healthcare services and reduced rates of psychological distress amongst TNB individuals. PCPs could provide better support through advocating on behalf of TNB patients, having more communication with their local TNB community and seeking access to guidelines and ongoing research. In addition, support should be provided directly to TNB patients through all stages of GAHC provision. There was a generalised desire for more primary care-led services to initiate and follow-up on gender-affirming treatments, as well as having more TNB representatives working in clinics.

### Comparison with existing literature

Cisnormativity at a societal and interpersonal level isolates TNB people, especially if they are marginalised in multiple ways, for example, being TNB and also disabled, neurodivergent, working class or a person of colour. Conforming to gendered stereotypes to be validated by primarily cisgender PCPs creates a culture of uncertainty, fear, and exhaustion in navigating primary care spaces. This is corroborated by previous research exploring barriers to healthcare access for TNB people, suggesting a high prevalence of negative healthcare encounters across services [6,16,39]. Participants who had had positive encounters were often surprised, speaking to the generalised negative perception of primary care, adding to the issue of healthcare avoidance based on lived, or anticipated, discrimination.

On an individual level, regarding GAHC, PCPs could provide further support for TNB people, for example, through prescribing bridging HRT, as detailed in guidelines developed by the UK Royal College of Psychiatrists [40]. However, in practice, participants requesting this were often refused by PCPs on personal grounds or due to lack of knowledge. Research exploring PCP perspectives on TNB healthcare further demonstrates a lack of knowledge and confidence on a structural, educational, and cultural level [5,41]. This extends to consultations unrelated to accessing GAHC, such as knowledge around cancer screening and monitoring of physical health conditions. Changes to medical school curricula to include TNB healthcare needs have been shown to improve clinical knowledge and attitudes towards TNB patients [42].

In the UK, this would require structural changes that redefine the role of primary care in trans-specific healthcare delivery. There is precedent for such change; the Republic of Ireland adopted a de-medicalised self-declarative system for gender recognition in 2015 [43]. Moreover, the World Health Organisation declassified being transgender as a mental or behavioural disorder in 2019, signifying a global change in the way being TNB is perceived [1]. Arguably, the reliance on psychiatric assessment in the UK perpetuates outdated assumptions that being TNB is a mental illness. However, changes to UK policy following consultation with over 100,000 respondents were halted, widely criticised by charities such as Stonewall and Mermaids [44,45].

### Strengths and limitations

To our knowledge, this is the first systematic review focusing on TNB people’s experiences of primary care. Many participants in the included studies were white and/or transgender men or women, with fewer nonbinary participants. There was limited focus on the intersection between different identities and how they influenced experiences of primary care.

For this review, there were challenges in defining primary care globally across different healthcare services; therefore the inclusion criteria were kept broad to ensure as much data was collected that was still within the remit of our research question. Two reviewers conducted the data collection and quality appraisal but only one reviewer completed the data synthesis, so there may be bias on the interpretation of findings.

Most studies were qualitative, from high-income countries; therefore there may not be sufficient data to generalise these findings.

### Implications for clinicians

The evidence from this review highlights both positive and negative experiences within primary care and where changes can be implemented.

PCPs can advocate for reform on a local and structural level in restructuring health policy, practice policy and medical education. International frameworks that may be useful include guidance published by the World Professional Association for Transgender Health and the Equality Challenge Unit, which outlines an overview of equality legislation, promoting TNB inclusive policies (including identifying documentation) and guidelines on cultivating a safe inclusive environment, which could be adapted for primary care settings in and outside of the UK [1,46]. It may be challenging within cisnormative cultures for PCPs to navigate and implement change in training programmes and medical practice. Local TNB communities or charities should be consulted in increasing accessibility and in the co-development of practice policy.

### Areas for future research

Recommendations implemented to improve gender-affirming primary care services can be evaluated, to add to the evidence available exploring TNB people’s experiences of primary care. There needs to be an extended effort to include the voices of nonbinary people and TNB people of colour that utilises an intersectional framework and to explore the experiences of TNB people of other demographics. There needs to be research as to facilitators and barriers for PCPs in providing and supporting access to GAHC to add to the evidence of how to implement structural change effectively. Future research should employ quantitative or mixed method designs to gather more objective data to strengthen the findings of existing qualitative research. It would be useful to evaluate existing or future pilots integrating primary care with GAHC provision and its impact on positive health outcomes.

## Conclusion

TNB people reported mixed experiences of primary care encounters. On a systemic level, there was a generally negative perception of primary care based on TNB people’s experiences navigating healthcare systems across high-income contexts. However, positive healthcare encounters and engagement with services were reported on a local level. There were variations in the consistency and quality of care and in navigating expectations of cisnormative PCPs and inaccessible healthcare systems. Multifaceted interventions that incorporate increasing PCP education and address areas such as improving the physical environment should be undertaken.

Challenges remain in reducing medical gatekeeping due to the continued pathologising of TNB identities. As such, there should be continued efforts to advocate for a reform to the GRA and the way GAHC is accessed in the UK.

## Supplementary Material

Supplemental Material

## Data Availability

The data underlying this article are available from the corresponding author on reasonable request.
